# Analysis of Surface Topography, Dimensional and Geometric Deviations, and Biocidal Properties of 3D Prints Made of Thermoplastic-Based Composites

**DOI:** 10.3390/ma19010129

**Published:** 2025-12-30

**Authors:** Urszula Kmiecik-Sołtysiak, Paweł Szczygieł, Dagmara Michta, Katarzyna Gałczyńska

**Affiliations:** 1 Faculty of Mechatronics and Mechanical Engineering, Kielce University of Technology, al. Tysiąclecia Państwa Polskiego 7, 25-314 Kielce, Poland; pszczygiel@tu.kielce.pl; 2 Faculty of Management and Computer Modelling, Kielce University of Technology, al. Tysiąclecia Państwa Polskiego 7, 25-314 Kielce, Poland; dmichta@tu.kielce.pl; 3 Institute of Biology, Jan Kochanowski University of Kielce, 7 Uniwersytecka Street, 25-406 Kielce, Poland; katarzyna.galczynska@ujk.edu.pl; 4 Central Office of Measures, Elektoralna 2 Street, 00-139 Warsaw, Poland

**Keywords:** FDM, PLA, coordinate measuring machine, nanomeasuring machine

## Abstract

This study evaluated the properties of two commercial filaments intended for medical and sterile applications: PLACTIVE (Copper 3D, Santiago, Chile) and CPE ANTIBAC (Fiberlogy, Brzezie, Poland). The aim of the research was to compare the dimensional accuracy, repeatability of the fused deposition modeling (FDM) 3D printing process, and the antibacterial properties of the samples using standardized procedures. Four types of samples were manufactured: geometrically differentiated specimens for metrological measurements (S1); cylinders with a diameter of 15 mm and a height of 40 mm for assessing process repeatability (S2); rectangular specimens measuring 40 × 40 × 2 mm for surface topography analysis (S3); and rectangular samples measuring 20 × 20 × 2 mm for biocidal property evaluation (S4). The results demonstrated that PLACTIVE samples exhibited higher dimensional conformity with nominal values and lower variability of diameters than CPE ANTIBAC samples, which may be associated with greater process stability. For both materials, the *PSm* parameter was correlated with layer height only in the 90° printing orientation. Surface topography analysis showed that increasing the layer height from 0.08 mm to 0.20 mm led to a significant rise in *Rsm*, *Ra*, and *Sa* values, indicating deterioration in the reproduction of micro-irregularities and increased spatial differentiation of the surface. For PLACTIVE samples, a tendency toward more convex structures with positive *Rsk* values and moderate kurtosis (*Rku*) was observed, suggesting uniform plasticization and stable interlayer bonding, particularly at the 0° orientation. In contrast, CPE ANTIBAC samples (especially those printed at 90°) were characterized by higher *Ra* and *Sa* values and negative skewness (*Rsk*), indicating valley-dominated, sharper surface morphology resulting from different rheological behavior and faster solidification of the material. PLACTIVE samples did not exhibit antibacterial properties against *Escherichia coli* (*E. coli*), while for *Staphylococcus aureus* (*S. aureus*), the activity was independent of printing direction and layer height. The CPE ANTIBAC material showed antibacterial effects against both tested strains in approximately 50% of the samples. The findings provide insights into the relationships between material type, printing orientation, and process parameters in shaping the dimensional and biocidal properties of FDM filaments.

## 1. Introduction

Additive manufacturing (AM), commonly referred to as 3D printing, has become one of the most rapidly developing technologies in modern materials engineering and biomedical applications. Among the various AM techniques, fused deposition modeling (FDM) stands out due to its accessibility, cost-effectiveness, and versatility in processing a wide range of thermoplastic polymers. The method enables the layer-by-layer fabrication of complex geometries directly from digital models, which is particularly beneficial in medical applications where patient-specific customization and rapid prototyping are of great importance. Consequently, FDM has been increasingly applied in the production of anatomical models, surgical guides, orthoses, and implant prototypes. Three-dimensional printing, also referred to as additive manufacturing (AM), consists of the additive formation of physical objects by depositing successive layers of material in accordance with the geometry defined by a digital spatial model or a computer-aided design (CAD) project. This process fundamentally differs from subtractive methods based on material removal. Through the precise dosing of material exclusively in regions required by the AM model, it considerably reduces raw-material waste and enables the fabrication of structures with high geometric complexity, which are difficult or impossible to obtain using conventional manufacturing techniques. Within additive manufacturing technologies, seven principal categories of processes are distinguished: material extrusion (including fused deposition modeling–FDM and fused filament fabrication–FFF), vat photopolymerization: stereolithography and digital light processing (SLA, DLP), powder bed fusion (selective laser melting–SLM, direct metal laser sintering–DMLS), material jetting, binder jetting, directed energy deposition (DED), and sheet lamination: laminated object manufacturing (LOM) [1].

3D printing is widely used across many industrial sectors, such as construction, aerospace, automotive engineering, biomedicine, and consumer goods manufacturing. In construction, AM technology supports sustainable development by reducing material waste, enabling the use of more environmentally friendly raw materials, and significantly shortening project execution times, which in turn lowers production costs [2]. In biomedicine, 3D bioprinting is developing particularly rapidly. It uses additive techniques to fabricate scaffolds incorporating living cells, biomaterials, and bioactive molecules. This enables the production of personalized implants, prosthetics, and surgical guides precisely tailored to a patient’s anatomy. Bioprinting also makes it possible to create tissue structures and organoids for regenerative medicine applications [3]. 3D printing also has numerous applications in medical education [4], allowing for the production of realistic anatomical and simulation models used in procedural training. In dentistry, AM technologies support the fabrication of precise prosthetic restorations and surgical guides [5]. Meanwhile, in the aerospace and automotive sectors, AM is used to manufacture lightweight, high-strength components, contributing to mass reduction and improved energy efficiency in transportation systems [1].

Due to the growing role of additive manufacturing technologies in medicine, the selection of appropriate biocompatible materials is of particular importance, as it enables the safe and effective production of implants, scaffolds, and other structures used in biomedical engineering. Among the commonly used biocompatible materials in 3D printing are polylactic acid (PLA), polycaprolactone (PCL), poly lactic-*co*-glycolic acid (PLGA), andpolyether ether ketone(PEEK). Owing to their biodegradability, controlled degradation, flexibility, or high mechanical strength, these materials can be used in the fabrication of scaffolds, prostheses, and implants. Their properties can be further modified by incorporating additives such as copper nanoparticles, which exhibit antibacterial activity. Despite their broad range of applications, these materials are associated with several challenges, including the risk of cytotoxicity resulting from residual monomers formed during incomplete polymerization [5].

Despite its potential, the use of conventional FDM polymers in biomedical applications is limited by insufficient biological activity and susceptibility to bacterial colonization. This issue is particularly relevant in the medical environment, where the risk of infection associated with contact surfaces and implantable devices remains a serious challenge. To address this, recent research has focused on developing polymeric materials with intrinsic antimicrobial properties, achieved through the incorporation of bioactive additives such as metallic nanoparticles, including silver (Ag) and copper (Cu). These metals are well known for their broad-spectrum antimicrobial activity, durability, and stability, and their incorporation into polymer matrices offers an effective strategy to inhibit bacterial growth without significantly compromising mechanical or printing properties.In this context, new commercial materials dedicated to medical and sterile applications have emerged, including PLACTIVE™ (Copper3D, Chile), a polylactic acid (PLA)-based filament modified with copper nanoparticles, and CPE ANTIBAC (Fiberlogy, Poland), a copolyester-based filament containing silver-based additives. Both materials are designed to combine printability typical for FDM processes with enhanced antimicrobial activity, making them promising candidates for use in environments requiring reduced microbial contamination, such as hospital surfaces, diagnostic equipment, or personalized medical tools.

However, despite the growing availability of such materials, systematic studies comparing their dimensional accuracy, process repeatability, and antibacterial effectiveness under standardized testing conditions remain limited. Understanding the relationship between material composition, process parameters, and the resulting functional properties is essential for validating their potential use in clinical or laboratory environments [6].

Measurements of components manufactured using the Fused Deposition Modeling (FDM) process are characterized by metrological challenges. FDM, an additive manufacturing technology based on material extrusion, produces parts layer by layer. Components fabricated using this method exhibit higher surface roughness parameters and greater deviations from nominal dimensions compared to parts produced using Selective Laser Sintering (SLS). In one reference study involving artifacts manufactured using both FDM and SLS, the average surface roughness parameter *Rz* was measured as 96.63 µm for FDM and 48.5 µm for SLS [7,8]. The surface roughness characteristics of FDM-manufactured components are one of the key factors contributing to increased measurement uncertainty when using coordinate measuring machines (CMMs).

The ability to maintain nominal dimensions and specified geometric tolerances in FDM-fabricated components depends on thermal shrinkage and the anisotropic nature of the process (anisotropic deposition effects). Larger components tend to exhibit smaller geometric deviations due to lower sensitivity to thermal shrinkage and material flow fluctuations, whereas smaller parts show anisotropic distortions resulting from the directional layer-by-layer structure [9]. Deviations from nominal values in the X-axis reported in [9] were negative (shrinkage), while positive values (expansion) dominated in the Y and Z directions. This phenomenon confirms the non-uniform nature of material cooling and crystallization in the FDM process.

Research on dimensional and geometric accuracy, as well as surface topography of 3D-printed components, requires advanced measurement techniques, including both contact and optical methods. Proper selection of parameters characterizing the surface, along with understanding how process parameters influence the final quality of printed components, is crucial.

Dimensional and shape accuracy, as well as surface texture characteristics of additively manufactured models, have been extensively documented in the scientific literature [10,11,12,13,14]. The authors of [10] presented results concerning the geometric accuracy of cylindrical gear teeth manufactured by the DMLS (direct metal laser sintering) method. The authors of [11] presented a comprehensive literature analysis regarding surface topography measurements for additively manufactured metal components. The surfaces of typical metal parts produced by powder-based methods are highly irregular and characterized by sharp irregularities. The study found that the most commonly used measurement technology is profile measurement using contact instruments. The most frequently utilized parameter for surface evaluation is the *Ra* parameter, the arithmetic mean deviation of the assessed profile. Another study [12] emphasized that components manufactured by AM methods are characterized by various internal defects, such as powder agglomeration, porosity, internal cracks, and thermal stresses, which significantly affect the quality, mechanical properties, and safety of final parts.

Research on dimensional accuracy and surface topography of components manufactured by additive methods demonstrates a comprehensive approach to product quality assessment. The authors of [13] conducted an analysis of wear in polymer molds produced by the PolyJet method, using a contact profilometer to measure surface topography.

Researchers emphasize the importance of reliable dimensional measurements in assessing the quality of 3D-printed elements, particularly using the FDM/FFF method. They point to the growing application of this technology in industry and the need for reliable control methods. The fundamental research problem is measurement uncertainty and the influence of various factors (e.g., technological seam) on result accuracy [14].

The aim of this study is therefore to compare the dimensional accuracy, process repeatability, and antibacterial properties of 3D-printed specimens fabricated using the FDM technique from two commercial filaments intended for medical and sterile applications: PLACTIVE™ and CPE ANTIBAC. The evaluation was conducted in accordance with normative procedures to ensure reproducibility and comparability of results. The findings are expected to provide insight into the practical potential and limitations of antimicrobial polymer filaments in medical additive manufacturing.

## 2. Materials and Methods

The samples were manufactured from two materials sold under the trade names PLACTIVE (Copper 3D, Santiago, Chile) and CPE ANTIBAC (Fiberlogy, Brzezie, Poland). According to the manufacturers’ declarations, these materials can be used for medical applications. They contain chemical modifiers with antibacterial properties (such as copper or silver) in a weight fraction of up to 1%. The full chemical composition of the materials is presented in Table 1.

In the case of studies on the biocidal properties of the PLACTIVE material, the manufacturer reports that they were conducted in two independent microbiological laboratories: SITU Biosciences in the United States and the Pontifical Catholic University of Valparaíso in Chile. The effects of the nanocomposite were analyzed on two bacterial strains: *Staphylococcus aureus* (MRSA) and *Escherichia coli* (DH5α).The results obtained in both institutions demonstrated very high effectiveness of the material even at the early stages of contact with microorganisms. After six hours of exposure, the number of colony-forming units (CFU) decreased by more than 95%, and after eight hours the reduction exceeded 98%. After 24 h, an almost complete elimination of bacteria was observed, surpassing 99.99% of the original population. Such high biocidal efficiency was attributed to the presence of the copper-based nanofiller, which deactivates a broad spectrum of microorganisms by damaging cell membranes and disrupting metabolic processes. The studies confirmed that the material maintains its mechanical and physical properties while effectively inhibiting bacterial growth on the printed surface [17].

The manufacturers provide data on the antimicrobial activity of the materials. In the case of CPE ANTIBAC, these tests were conducted in accordance with ISO 22196 [18]. Samples with a surface area of 16 cm^2^ were exposed to two reference bacterial strains: *Escherichia coli* (ATCC 8739) and *Staphylococcus aureus* (ATCC 6538). Each sample was inoculated with 0.4 cm^3^ of a bacterial suspension containing a specified number of cellsandthen covered with a sterile polyethylene film and incubated for 24 h. After incubation, the number of viable cells on the material surface was determined. The obtained results confirmed the strong biocidal properties of the material. For *E. coli*, complete elimination of bacteria was observed after 24 h, corresponding to 100% reduction and an antimicrobial activity level of 5.9 log. For *S. aureus*, the reduction in cell count was 92.4%, with an activity of 1.1 log. These results indicate that the presence of silver within the material structure effectively inhibits the growth of microorganisms, particularly Gram-negative bacteria [19].

The manufacturers report very high biocidal efficacy for both materials. PLACTIVE achieved near-complete bacterial elimination for all tested strains (>99.99%), while CPE ANTIBAC demonstrated full effectiveness against *E. coli* and slightly lower activity against *S. aureus*. Both materials can therefore be considered effective in limiting microbial growth, with differences primarily attributed to the type of active additive used.

The samples used in the studies presented in this article were made from materials in their factory-new state, in the form of 1.75 mm diameter filament spooled onto reels. The 3D models of the samples were designed using SOLIDWORKS 2023 (Dassault Systèmes, Vélizy-Villacoublay, France) and then saved in STL (Standard Tessellation Language) format. The technological parameters of the additive manufacturing process were set using Bambu Studio software v. 2.0.1.50 (Bambu Lab, Shenzhen, China) by importing the previously saved STL models.

**Figure 1:**
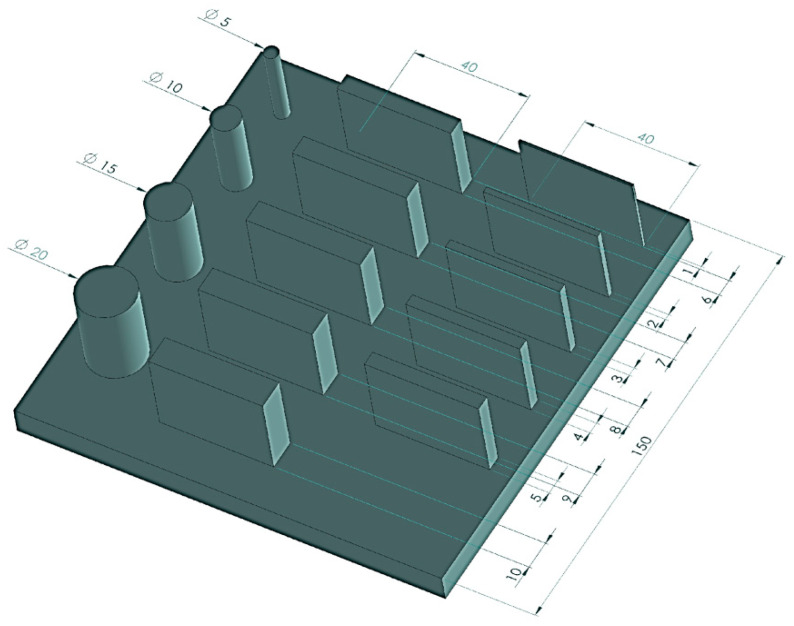
Dimensions of the S1-type sample.

The additive manufacturing process was carried out using a Bambu Lab X1C 3D printer (Bambu Lab, Shenzhen, China) employing FDM (fused deposition modeling) technology. The printer was equipped with a 0.4 mm diameter nozzle and a textured PEI build plate. Four types of samples were produced:

1. Samples intended for dimensional and geometric measurements with varied geometries (Figure 1), consisting of a set of cylinders of different diameters and cuboids of varying thicknesses. These allowed the assessment of dimensional and geometric accuracy, including cylindricity and flatness measurements—these samples were designated as S1.
2. Cylinders with a diameter of 15 mm and a height of 40 mm, measured to evaluate the repeatability of the 3D printing process—these samples were designated as S2.
3. Cuboid samples measuring 40 mm × 40 mm × 2 mm, used for surface topography measurements—S3.
4. Cuboid samples measuring 20 mm × 20 mm × 2 mm, used for biocidal property studies—these samples were designated as S4.

The technological parameters of the additive manufacturing process are presented in Table 2. For samples S1 and S2, the variable parameter was the layer height, set at two different levels. For samples S3 and S4, the variable parameters were orientation (placement of the samples on the 3D printer build platform) and layer height, also set at two different levels.

**Figure 2:**
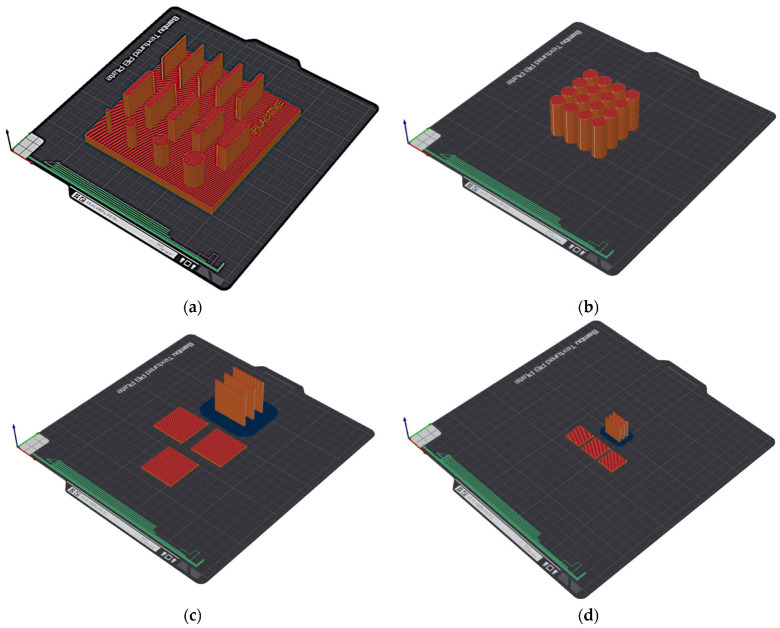
Arrangement of the samples on the 3D printer build platform: (**a**) S1, (**b**) S2, (**c**) S3, and (**d**) S4.

The samples were produced in varying numbers of repetitions, as shown in Table 3, along with their designations. The arrangement of the samples on the 3D printer build platform is shown in Figure 2.

**Figure 3:**
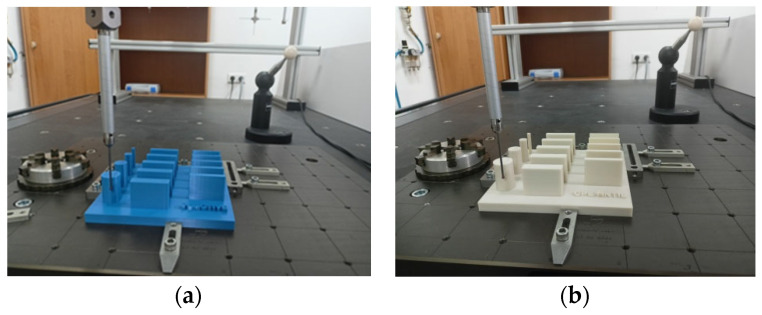
Sample (**a**) S1-1 and (**b**) S1-2 during CMM measurements.

**Figure 4:**
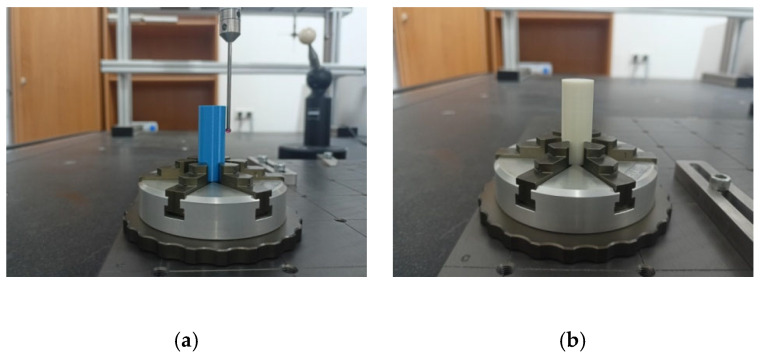
Sample (**a**) S2-1 and (**b**) S2-2 clamping during CMM measurements.

Distance, diameter, and geometric deviation measurements of S1 samples (Figure 3), as well as diameter measurements of S2 samples (Figure 4), were performed using a Zeiss Prismo Navigator coordinate measuring Machine (Zeiss, Oberkochen, Germany) equipped with Zeiss Calypso 2015 software v. 6.0.12 (Zeiss, Oberkochen, Germany). The device was equipped with a Zeiss VAST Gold active scanning probe head(Zeiss, Oberkochen, Germany), which, thanks to the Navigator system, enables variable-speed scanning, including high-speed scanning up to 300 mm/s. The use of an active scanning probe head allows for high accuracy in form deviation measurements. A measuring stylus with a 3 mm diameter ruby tip was used for measurements. For cylindricity deviation measurements (performed in a spiral strategy), 1000 measurement points were recorded for each measurement. The scanning speed was 10 mm/s. A Gauss filter with a range of 1–50 undulations per revolution (UPR) was applied. For flatness deviation measurements, each plane was scanned using a polyline strategy, collecting 1000 points on each plane. Environmental conditions were strictly controlled, with temperature maintained within ±0.5 °C. The measuring range of the Zeiss Navigator coordinate measuring Machine is 900 mm × 1200 mm × 700 mm. The maximum permissible error of the Prismo Navigator measuring instrument for linear dimensions is 0.9 µm + L/350 µm (where L is the measured length expressed in meters). The maximum permissible error for roundness deviation measurements is 1 µm.

**Figure 5:**
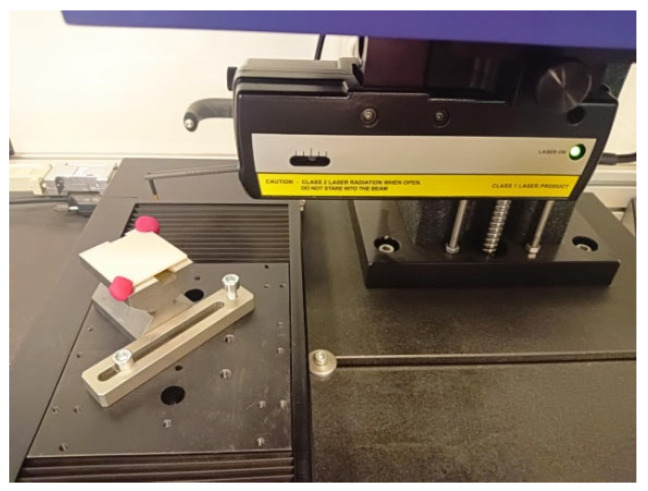
Sample S3-1-S placed on the measuring table of the Form Talysurf PGI 1200 contact profilometer (Taylor Hobson, Leicester, United Kingdom).

**Figure 6:**
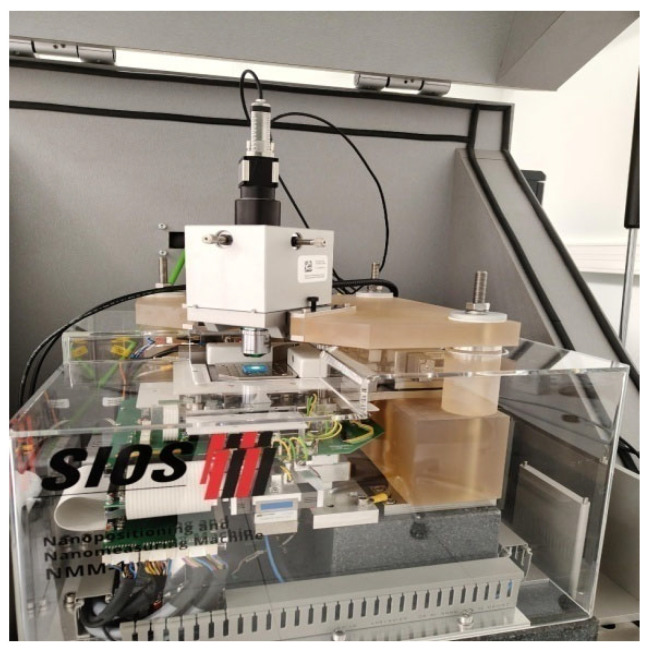
Sample S3-1-M placed on the SIOS Nanomeasuring Machine table (SIOS Meßtechnik GmbH, Ilmenau, Germany).

Surface topography analysis of S3 samples (Figure 5 and Figure 6) was performed using the WLI (white light interferometer) optical head of the SIOS Nanomeasuring Machine (SIOS Meßtechnik GmbH, Ilmenau, Germany) and the Form Talysurf PGI 1200 contact profilometer (Taylor Hobson, Leicester, United Kingdom) using Mountains 10 software v. 5.1.1.5374 (Digital Surf Headquarters, Besançon, France). The resolution of the Form Talysurf PGI profilometer in the Z-axis is 0.8 µm, and in the X-axis it is 0.125 µm. The contact profilometer was equipped with a measuring tip with a stylus angle of 60° and a tip radius of 2 µm. Measurements using the optical head were performed with a 20× magnification objective. The advantages of coherence scanning interferometry (white light sensor, WLI optical head) include the absence of interaction between the measuring instrument and the measured surface, as well as low inherent noise (*Rq* < 80 pm). The contact measurement instrument (Form Talysurf PGI) with an interferometric probe head is characterized by a low noise value of 1.99 µm. In coherence scanning interferometry, measurement parameters depend directly on the magnification of the objective used. For objectives with magnifications of 10×, 20×, and 50×, both pixel spacing and resolution, as well as field of view, change. At lower magnification (10×), pixel spacing was approximately 0.74 µm, and lateral resolution was 1.48 µm, enabling observation of a larger sample area with a field of view of 740 × 740 µm^2^. Increasing magnification to 20× improves measurement accuracy—pixel spacing decreases to 0.37 µm and lateral resolution to 0.90 µm, but the field of view is reduced to 370 × 370 µm^2^. The highest magnification (50×) provides the greatest measurement precision, with pixel spacing of 0.148 µm and lateral resolution of 0.65 µm, but the recorded sample area is then smallest—148 × 148 µm^2^. Non-contact optical surface scanning enables low positioning noise and a large measurement range, achieved through a 5 mm z-axis positioning capability.

All measurements were performed under controlled laboratory conditions, at an ambient temperature of 20 ± 0.5 °C and relative humidity of 45–55%, by a single operator.

The assessment of antibacterial properties was performed against *Escherichia coli* and *Staphylococcus aureus* using the colony-forming unit assay (CFU) method in accordance with ISO 22196. S4 samples were placed in a 6-well plate, then inoculated with 200 µL of bacterial suspension at a concentration of 10^5^ CFU in 1:500 diluted LB medium and covered with a film. The plates were incubated for 24 h at 35 °C. After incubation, the bacteria were rinsed off, serial dilutions were prepared in physiological saline (0.9% NaCl), and CFU were determined using the pour plate method. The results are expressed as Log_10_ CFU/mL.

## 3. Results

**Figure 7:**
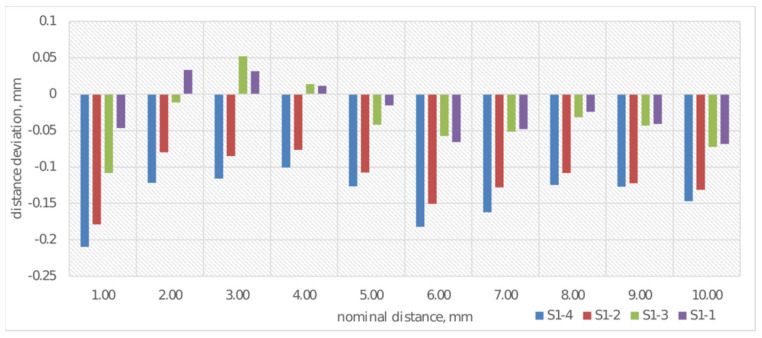
Average deviations from the nominal value for distance measurement for S1-1–S1-4.

After performing measurements of samples S1-1 through S1-4 using the coordinate measuring machine, an analysis of the obtained results was conducted. Each measurement was performed three times, and the presented graphs contain average values from the obtained results. Deviations from the nominal value of distances for cuboids of different thicknesses were analyzed, with results shown in Figure 7. The measurement of deviation from the nominal distance was performed by determining two parallel planes for each of the examined cuboids.

**Figure 8:**
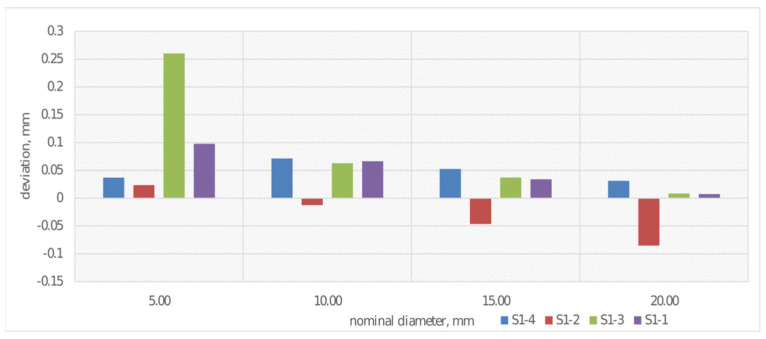
Average deviations from the nominal value for diameter measurement for S1-1–S1-4.

In external biomedical applications, cylindrical 3D-printed structures are used in, among other things, limb prostheses, orthoses, and rehabilitation support elements, where their shape ensures stability and even load distribution. The cylindrical form also allows for the creation of lightweight, ventilated, yet durable components that conform well to the patient’s body shape. Therefore, the study included an analysis of diameter dimensions. Using a coordinate measuring machine, the deviation from the nominal value for the diameters of each of the four rollers was determined. The results are shown in Figure 8.

**Figure 9:**
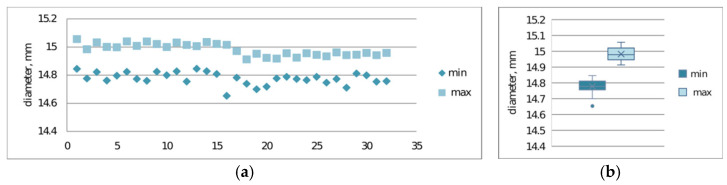
Print repeatability study considering maximum and minimum diameter measurements of S2-1 made from PLACTIVE material: (**a**) plot of maximum and minimum print diameters; (**b**) box-and-whisker plot.

**Figure 10:**
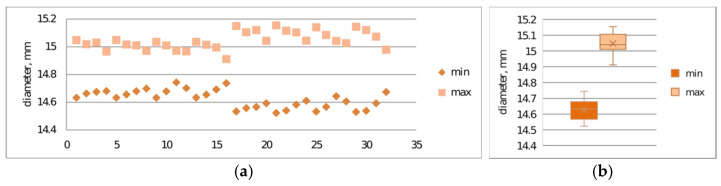
Print repeatability study considering maximum and minimum diameter measurements of printed S2-3 made from CPE ANTIBAC material: (**a**) plot of maximum and minimum print diameters; (**b**) box-and-whisker plot.

Verification of print repeatability constitutes a key element in assessing the stability of the additive process, enabling determination of dimensional and mechanical variability between successive samples. Repeatability analysis allows for the identification of systematic and random deviations resulting from technological parameters, thermal properties of the material, or dynamics of the printer’s mechatronic system operation.The repeatability of prints was examined. Figure 9a,b and Figure 10a,b present the results of maximum and minimum diameter measurements for samples S2-1 and S2-2.

**Figure 11:**
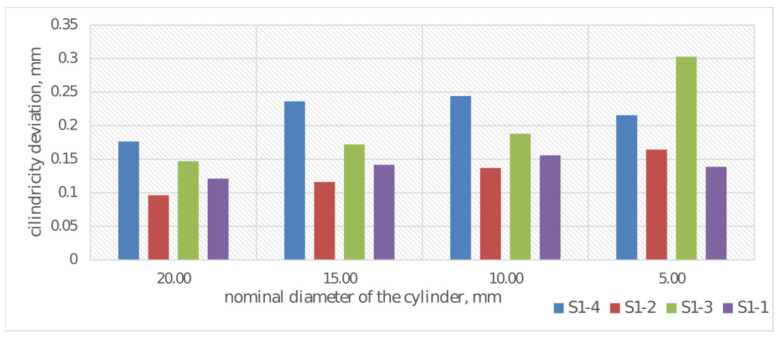
Cylindricity deviations for diameter measurement for samples S1-1–S1-4.

In addition to dimensional analysis of printed samples S1-1 through S1-4, geometric deviation analysis was also performed. Average cylindricity deviations were determined for each cylinder. The results of deviation measurements are presented in Figure 11.

**Figure 12:**
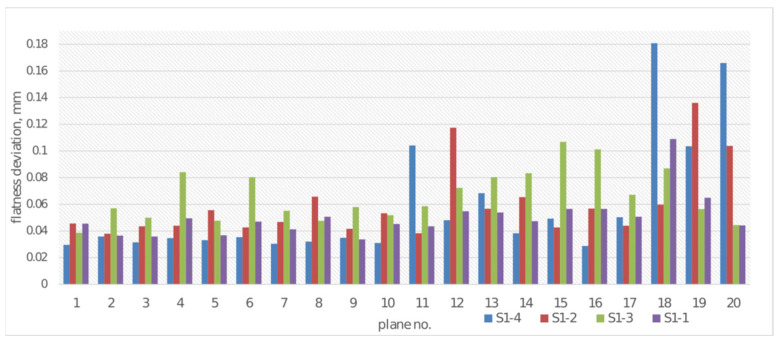
Flatness deviations for plane measurement for samples S1-1–S1-4.

The planes examined using the coordinate measuring machine were also used to determine the flatness deviation of each of the two planes of each cuboid on samples S1-1 through S1-4. Each examined S1 sample contained 10 cuboids with thicknesses ranging from 1 to 10 mm, in 1 mm increments. Planes 1 and 2 were determined on the cuboid with a thickness of 10.00 mm, planes 3 and 4 on the 9.00 mm cuboid, etc. The flatness deviation results are shown in Figure 12.

**Figure 13:**
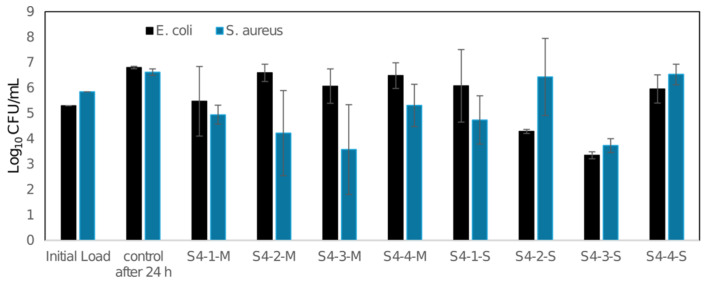
Log_10_ colony-forming unit (CFU) assays of *E. coli* and *S. aureus*.

An antibacterial analysis of the tested materials was conducted. Antibacterial property testing of the 3D-printed materials, conducted in accordance with ISO 22196, demonstrated that the PLA material (M) did not exhibit any antibacterial activity against *Escherichia coli* in any of the tested variants. However, variants S4-2-M and S4-3-M showed antibacterial activity against Staphylococcus aureus, with a reduction of 90% and 99%, respectively. In contrast, materials S4-2-S and S4-3-S were antibacterial against *E. coli*, with reductions of 90% and 99%, respectively. Regarding *S. aureus*, antibacterial properties were observed for S4-1-S and S4-3-S, with reductions of 90% and 99%, respectively (Figure 13).

**Figure 14:**
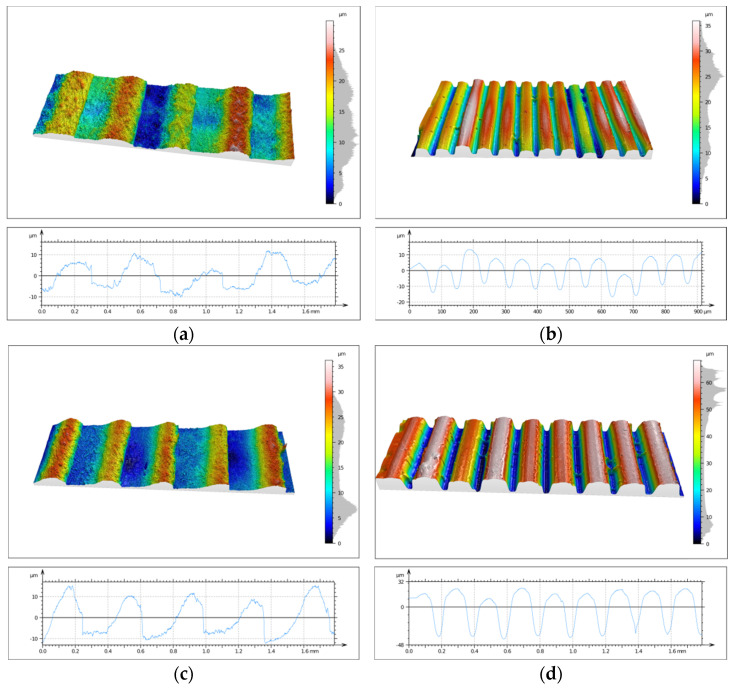
Isometric image of sample S3 surface: (**a**) 1M, (**b**) 2M, (**c**) 3M, and (**d**) 4M.

**Figure 15:**
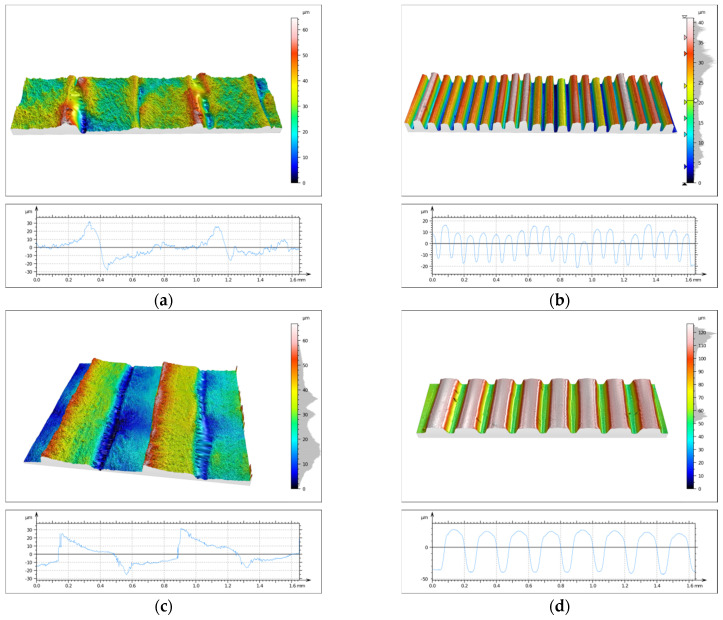
Isometric image of sample S3 surface: (**a**) 1S, (**b**) 2S, (**c**) 3S, and (**d**) 4S.

One of the main objectives of the conducted research was to evaluate the topography state of prints made from two different biocidal materials. This analysis was performed using the SIOS Nanomeasuring Machine. The *PSm* parameter, defined as the mean spacing of the primary profile, reflects the degree of surface irregularity produced by 3D printing. In the case of the 90° printing direction, this parameter is closely related to layer height, with the *PSm* value reflecting its magnitude. CPE ANTIBAC material is characterized by slightly higher *PSm* values compared to PLACTIVE, which may result from differences in the structure and thermal characteristics of both filaments. The results of *PSm* parameter measurements are presented in Table 4. Determination of the primary profile parameter was performed using the WLI white light head of the SIOS Nanomeasuring Machine. The obtained isometric images are shown in Figure 14 and Figure 15.

Using the Form Talysurf PGI roughness profilometer, mean values of roughness profile parameters (*Ra*, *Rsm*, *Rku*, and *Rsk*) and the surface topography parameter *Sa* were determined. The measurement results are presented in Table 5.

## 4. Discussion

Analysis of deviations from nominal values of cuboid thicknesses (Figure 7) indicates that the deviations show a tendency toward negative values, which indicates systematic underestimation of actual thicknesses relative to nominal values. Sample S1-4 (print made from CPE ANTIBAC material with a print layer height of 0.20 mm) is characterized by the largest deviation amplitude, which may suggest reduced print stability. Samples made from PLACTIVE material (S1-1 and S1-3) exhibit the smallest deviation values, which implies their greater printing precision. It was observed that the print made from PLACTIVE material with a print layer height of 0.08 mm was characterized by deviations closest to the nominal thickness values of the cuboids.

In the case of measuring deviation from nominal cylinder diameter values (Figure 8), the greatest discrepancy was observed for the 5 mm diameter, especially for sample S1-3. For cylinders made from PLACTIVE material with diameters of 10 and 20 mm, deviations are significantly more stable and do not reach 0.05 mm, which suggests improvement in measurement accuracy with increasing diameter. Cylinders made from CPE ANTIBAC material with a layer height of 0.20 mm (sample S1-2) exhibit the largest negative deviations at diameters of 15 and 20 mm. Overall, the results suggest that as the examined cylinder diameter increases, deviations from nominal values decrease.

The conducted studies also allowed for assessment of print repeatability (Figure 9 and Figure 10). Samples made from CPE ANTIBAC material are characterized by greater diameter measurement scatter compared to PLACTIVE samples, which indicates lower printing process stability. In the case of cylinders made from PLACTIVE material, diameter values are characterized by a clearly smaller standard deviation on the order of 0.04 mm compared to those made from CPE ANTIBAC material: 0.06 mm.

Analysis of geometric deviations in the assessment of cylindricity deviation indicates a significant increase in cylindricity deviation with decreasing nominal cylinder diameter. The highest cylindricity deviation values are exhibited by cylinders manufactured using a print layer height of 0.20 mm and CPE ANTIBAC material. Cylinders in samples S1-2 and S1-1 exhibit lower and more stable cylindricity deviation values. Overall, the observed trend (Figure 11) indicates increasing difficulty in maintaining shape accuracy for small diameters, which may result from technological limitations.

Flatness deviations exhibit considerable variability (Figure 12), with sample S1-4 (CPE ANTIBAC., 0.20 mm) achieving the highest values in planes 18 and 20, exceeding 0.16 mm. Samples manufactured with greater layer height (0.20 mm)—S1-3 and S1-4—exhibit higher deviation values compared to samples with a layer height of 0.08 mm (S1-1 and S1-2), which suggests that thicker print layers contribute to increased surface irregularities. CPE ANTIBAC material at a layer height of 0.20 mm (S1-4) is characterized by the most extreme deviation values, while the same material at 0.08 mm (S1-2) exhibits relatively stable and low values, rarely exceeding 0.06 mm. Sample S1-3 (PLACTIVE, 0.20 mm) shows moderate deviation values with several local maxima in planes 12 and 15, which indicates better dimensional stability of this material compared to CPE ANTIBAC at the same layer height. All samples exhibit the smallest deviation values in the initial planes (for cuboids with thicknesses of 8–10 mm), which may suggest better adhesion and printing process stability in the initial phase.

Analysis of surface topography, its parameters, and roughness profile parameters is extremely important and provides much valuable information. Measurements of these parameters are significant due to the influence of surface irregularities and topographic defects on stresses. Roughness parameters directly correlate with material fatigue resistance, and anisotropy resulting from print direction affects differences in strength. Quantification of topography parameters constitutes an objective criterion for print quality assessment, enabling comparison of different materials, technologies, and process settings. The primary profile parameter *PSm*, defined as the mean spacing of the primary profile, reflects the degree of surface irregularity produced by 3D printing. In the case of the 90° print direction, this parameter is closely related to print layer height, with the *PSm* value reflecting its magnitude. For the 0° print direction, CPE ANTIBAC material is characterized by slightly higher *PSm* values compared to PLACTIVE, which may result from differences in the structure and thermal characteristics of both filaments. The *Rsk* parameter is called the skewness coefficient and is the third-order moment of the amplitude distribution curve determined over the elementary section length. The *Rku* parameter is the kurtosis of the roughness profile. The *Rsk* and *Rku* parameters indicate significant differences in the character of irregularity distribution between individual samples. Positive *Rsk* values (e.g., for sample S3-1-M with 0° print direction and 0.08 mm layer height and S3-3-M with 0° print direction and 0.2 mm layer height) indicate that these surfaces have a predominance of high peaks. *Rku* values reflect the degree of “sharpness” or “flatness” of the profile. Most samples are characterized by values in the 2–3 range, which suggests a distribution close to normal, while sample S3-1-S stands out with a significantly higher value of *Rku* = 8.27, which indicates the presence of exceptionally sharp peaks or deep valleys in the surface structure. The compilation of parameters thus allows us to conclude that the examined surfaces differ not only in average roughness but also in profile shape and symmetry. Analysis of *Rsk* and *Rku* values enables a better understanding of the functional properties of surfaces—including their potential behavior in friction and wear processes. CPE ANTIBAC material at a layer height of 0.20 mm and 0° orientation (S3-3-S) exhibits a *Ra* parameter = 26.26 μm, while PLACTIVE at the same printing parameters (S3-3-M) achieves *Ra* = 4.94 μm, which indicates poorer surface quality for CPE ANTIBAC. It appears reasonable that the rheological and crystallization properties of CPE ANTIBAC require optimization of printing parameters, particularly for printing in 0° orientation and 0.20 mm layer height. The smallest values of *Ra* and *Sa* parameters were exhibited by prints made from PLACTIVE material with a 0.08 mm print layer in 0° orientation.

It is widely reported in the literature that the antibacterial activity of functional surfaces depends not only on the presence of an active ingredient, such as silver ions (Ag^+^) or copper ions (Cu^2+^/Cu^+^), but also on surface-related factors, including surface roughness parameters and anisotropy, resulting from the manufacturing process [20,21,22]. In polymeric materials modified with ion-releasing antibacterial agents, increased surface roughness parameters can improve the contact between the material surface and bacterial cells and promote the local availability of metal ions at the material–environment interface. Such effects are associated with cell membrane damage, protein dysfunction, and oxidative stress in bacterial cells, ultimately leading to reduced cell viability [20,23,24]. At the same time, surface topography strongly influences bacterial adhesion; therefore, the interpretation of antibacterial activity must take into account both the inactivation mechanisms and possible differences in bacterial adhesion related to the surface geometry [25]. The CPE ANTIBAC material, whose antibacterial activity is related to the presence and release of silver ions (Ag^+^), was characterized by higher surface roughness parameters (*Ra* = 26.26 µm for 0° orientation and 0.20 mm layer height) and antibacterial activity against *Escherichia coli* and *Staphylococcus aureus*. According to literature reports, Ag^+^-based systems, including silver ions immobilized in or released from inorganic carriers such as zeolites, exhibit a broad spectrum of antibacterial activity, with the scale and rate of this effect depending on environmental conditions and ion availability [26,27,28]. In contrast, the PLACTIVE material, whose antibacterial activity is due to its copper-containing component and the availability of copper ions (Cu^2+^/Cu^+^), was characterized by significantly lower roughness values (*Ra* = 4.94 µm at identical printing parameters as the CPE ANTIBAC sample mentioned in this paragraph). In this study, it demonstrated antibacterial activity only against *Staphylococcus aureus*. This observation is consistent with previous studies on copper-modified polymeric materials intended for additive manufacturing, which demonstrated that antibacterial activity depends on processing conditions, surface exposure, and ion release characteristics [29,30], as well as with reports describing the contact and ionic mechanisms of copper-based antimicrobial surfaces [21,22]. The presented results indicate that surface roughness can act as a modulating (enhancing) factor in antibacterial activity, but it is not sufficient in itself to predict antibacterial efficacy. The ultimate antibacterial effect results from the synergy between surface topography (shaped by printing parameters), the availability and release of metal ions from the surface, and the susceptibility of a given bacterial strain to specific antibacterial mechanisms.

Based on the obtained results, three principal directions for further investigation have been identified. First, the optimization of process parameters for CPE ANTIBAC should be pursued through detailed rheological analyses and crystallization kinetics studies, with the aim of enhancing dimensional stability and controlling microstructural development during processing. Second, it is essential to elucidate the mechanisms governing the release of biocidal compounds from the polymer matrix, as well as to establish quantitative correlations between surface topography parameters and bacterial adhesion, thereby providing insights into the antimicrobial performance of the material. Third, a comprehensive characterization of the mechanical properties is required, taking into account structural anisotropy, fatigue resistance, and the impact of surface defects on local stress concentrations, in order to predict material behavior under complex loading conditions and ensure long-term reliability in practical applications.

## 5. Conclusions

Analysis of dimensional deviations, surface topography, and antibacterial properties of 3D prints allowed for the formulation of the following conclusions:

- PLACTIVE filament demonstrated higher dimensional precision and better process repeatability compared to CPE ANTIBAC filament, with the smallest deviations from nominal values obtained for a layer height of 0.08 mm in 0° print orientation. From a microbiological perspective, PLACTIVE did not exhibit antibacterial activity against *Escherichia coli*, while antibacterial effects against *Staphylococcus aureus* were observed only for selected printing conditions.
- Cylindricity and flatness deviations increased with decreasing nominal diameter of elements and increasing print layer height from 0.08 mm to 0.20 mm, which indicates limitations in the accuracy of reproducing small geometric structures in FDM technology.
- CPE ANTIBAC material in 0° orientation and at a print layer height of 0.20 mm was characterized by more than five times higher surface roughness (*Ra* = 26.26 μm) compared to PLACTIVE at identical printing parameters (*Ra* = 4.94 μm), which suggests different rheological properties and crystallization kinetics requiring optimization of process parameters. At the same time, CPE ANTIBAC demonstrated antibacterial activity against both *Escherichia coli* and *Staphylococcus aureus*; however, the effect was dependent on printing parameters and was not observed in all tested variants.

## Figures and Tables

**Table 1:** Chemicalcomposition of materials [15,16].

Component	Description	wt.%
**PLACTIVE**		
Polylactideresin	Base resin (biodegradable polymer)	>99
NanoCu PCZ001	Copper nanoadditives (antibacterial)	<1
**CPE ANTIBAC**		
Copolyester	Main structural component	>99
Zincoxide	Antibacterial additive improves UV resistance	<0.9
Silver	Component with strong biocidal properties	<0.1

**Table 2:** Additive manufacturing parameters.

Parameter	Value	Unit
Layer height	0.08 or 0.20	mm
Seam	Aligned	-
Wall loops	2	-
Infill pattern	Rectilinear	-
Infill density	100	%
Nozzle temperature	220 (PLACTIVE) 270 (CPE ANTIBAC)	°C
Bed temperature	50 (PLACTIVE) 110 (CPE ANTIBAC)	°C
Print speed	200 (PLACTIVE) 100 (CPE ANTIBAC)	mm/s

**Table 3:** Sample designation.

Sample Designation	Number of Repetitions	Layer Height	Material	Orientation
S1-1	1	0.08 mm	PLACTIVE	
S1-2	1	0.08 mm	CPE ANTIBAC	
S1-3	1	0.20 mm	PLACTIVE	
S1-4	1	0.20 mm	CPE ANTIBAC	
S2-1	32	0.08 mm	PLACTIVE	
S2-2	32	0.08 mm	CPE ANTIBAC	
S3-1-M	3	0.08 mm	PLACTIVE	0°
S3-2-M	3	0.08 mm	PLACTIVE	90°
S3-3-M	3	0.20 mm	PLACTIVE	0°
S3-4-M	3	0.20 mm	PLACTIVE	90°
S3-1-S	3	0.08 mm	CPE ANTIBAC	0°
S3-2-S	3	0.08 mm	CPE ANTIBAC	90°
S3-3-S	3	0.20 mm	CPE ANTIBAC	0°
S3-4-S	3	0.20 mm	CPE ANTIBAC	90°
S4-1-M	3	0.08 mm	PLACTIVE	0°
S4-2-M	3	0.08 mm	PLACTIVE	90°
S4-3-M	3	0.20 mm	PLACTIVE	0°
S4-4-M	3	0.20 mm	PLACTIVE	90°
S4-1-S	3	0.08 mm	CPE ANTIBAC	0°
S4-2-S	3	0.08 mm	CPE ANTIBAC	90°
S4-3-S	3	0.20 mm	CPE ANTIBAC	0°
S4-4-S	3	0.20 mm	CPE ANTIBAC	90°

**Table 4:** Values of the primary profile *PSm* parameter.

Sample Identification	Average Value *Psm*, mm	Standard Deviation Value *Psm*, mm
S3-1-M	0.325	0.029
S3-2-M	0.088	0.000
S3-3-M	0.375	0.002
S3-4-M	0.200	0.000
S3-1-S	0.176	0.042
S3-2-S	0.074	0.002
S3-3-S	0.501	0.127
S3-4-S	0.200	0.000

**Table 5:** Roughness profile parameters and surface topography parameters determined from isometric images of S3 samples obtained by contact measurement.

Sample Identification	Average Value *Rsm*, mm	Average Value *Ra*, µm	Average Value *Sa*, µm	Average Value *Rku*	Average Value *Rsk*
S3-1-M	0.267	2.76	3.66	4.35	0.70
S3-2-M	0.081	4.92	5.30	2.20	−0.54
S3-3-M	0.361	4.94	3.81	2.29	0.94
S3-4-M	0.199	13.36	13.58	2.30	−0.73
S3-1-S	0.342	10.30	14.76	8.27	−2.28
S3-2-S	0.081	5.80	5.98	2.20	−0.49
S3-3-S	0.750	26.26	26.55	2.27	−0.63
S3-4-S	0.199	13.75	13.95	2.31	−0.69
**Sample Identification**	**Standard Deviation Value** ***Rsm*, mm**	**Standard Deviation Value** ***Ra*, µm**	**Standard Deviation Value** ***Sa*, µm**	**Standard Deviation Value** ***Rku***	**Standard Deviation Value** ***Rsk***
S3-1-M	0.021	0.35	0.27	0.38	0.05
S3-2-M	0.000	0.01	0.18	0.01	0.01
S3-3-M	0.024	0.25	0.44	0.11	0.06
S3-4-M	0.000	0.03	0.42	0.01	0.00
S3-1-S	0.066	1.30	0.31	1.04	0.23
S3-2-S	0.001	0.02	0.29	0.01	0.01
S3-3-S	0.009	3.60	1.51	0.39	0.17
S3-4-S	0.000	0.02	0.21	0.02	0.01

## Data Availability

The original contributions presented in this study are included in the article. Further inquiries can be directed to the corresponding author.

## Abbreviations

The following abbreviations are used in this manuscript:

AM	Additive Manufacturing
CFU	Colony Forming Units
CMM	Coordinate Measuring Machine
CPE	Carbapenemase–Producing Enterobacterales
FDM	Fused Deposition Modeling
PLA	Polylactic Acid
SLS	Selective Laser Sintering
